# Etiologic Determinants and Characteristics of Diabetes in Haitian Youth (EDDHY Study)

**DOI:** 10.1155/pedi/9974561

**Published:** 2025-05-25

**Authors:** Eddy Jean-Baptiste, Philippe Larco, Julia E. Von Oettingen, Janelle A. Noble, Steven J. Mack, Ningyi Song, Harper R. N. Martin, Erik Rozemuller, Mark A. Atkinson, Denira Govender, Nancy Charles Larco, Graham D. Ogle

**Affiliations:** ^1^Fondation Haïtienne de Diabète et de Maladies Cardiovasculaires (FHADIMAC), Port-au-Prince, Haiti; ^2^Research Institute of the McGill University Health Centre, Montreal, Canada; ^3^Department of Endocrinology and Metabolism, Montreal Children's Hospital, Montreal, Canada; ^4^Children's Hospital Oakland Research Institute, Oakland, California, USA; ^5^Department of Pediatrics, University of California, San Francisco, Oakland, California, USA; ^6^Tongji Hospital, Tongji Medical College, Huazhong University of Science and Technology, Wuhan, China; ^7^GenDx, Genome Diagnostics B.V., Utrecht, Netherlands; ^8^Department of Pathology, Immunology and Laboratory Medicine, College of Medicine, Diabetes Institute, University of Florida, Gainesville, Florida, USA; ^9^Department of Pediatrics, College of Medicine, Diabetes Institute, University of Florida, Gainesville, Florida, USA; ^10^Life for a Child Program, Diabetes Australia, Glebe, New South Wales, Australia; ^11^Sydney Medical School, University of Sydney, Sydney, New South Wales, Australia

**Keywords:** autoimmunity, genetics, idiopathic type 1 diabetes, youth-onset diabetes

## Abstract

**Aims:** Published information on youth-onset diabetes in Haiti is scarce, with limited data available on diabetes autoimmunity and genetic susceptibility to the disease. We determined the anthropometric, metabolic, and immunological characteristics and human leukocyte antigen (HLA)-associated risks in patients with youth-onset diabetes.

**Methods:** One hundred and ten subjects with type 1 diabetes (T1D) aged <22 years and diagnosed for < 2 years were evaluated. Demographic and clinical information, as well as biochemical parameters, including blood glucose, hemoglobin A1c, fasting C-peptide (FCP), and T1D-associated autoantibodies, were assessed. DNA from 54 subjects and 66 controls was genotyped for classical HLA loci.

**Results:** Of the 110 patients, 54% were male. Onset age was 13.5 ± 4.2 years (range 2–21), and disease duration was 11.7 ± 8.1 months (range 0–24). Idiopathic T1D was found in 62 (56.4%) patients and was diagnosed at an older age than immune-mediated T1D (14.4 ± 3.5 years vs., 12.3 ± 4.8 years, *p*=0.01), with a higher BMI z-score in patients aged <14 years than in those aged ≥14 years (−0.29 ± 1.52 vs., −1.15 ± 1.18, *p*=0.01). No correlation was found between immune-mediated T1D and BMI z-score. Diabetic ketoacidosis was present at diagnosis in 18 (16.4%) patients. Zinc transporter 8 autoantibodies (ZnT8A) were marginally more common in younger patients. Low FCP levels were found in 71 (64.5%) patients. Thyroid peroxidase antibodies (TPO-Ab) and thyroglobulin antibodies (TG-Ab) were positive in 1.1% and 2.2% of the patients, respectively. The alleles *DRB1⁣*^*∗*^*03:01*, *DRB1⁣*^*∗*^*09:01*, *DQB1⁣*^*∗*^*02:01*, and *DQB1⁣*^*∗*^*02:02* showed a significant T1D risk, whereas *DRB1⁣*^*∗*^*08:04*, *DRB1⁣*^*∗*^*15:03*, and *DQB1⁣*^*∗*^*06:02* were protective. Three *DRB1~DQB1* haplotypes were strongly associated with T1D: *DRB1⁣*^*∗*^*03:01:01~DQB1⁣*^*∗*^*02:01:01*, *DRB1⁣*^*∗*^*09:01:02~DQB1⁣*^*∗*^*02:02:01*, both predisposing, and *DRB1⁣*^*∗*^*15:03:01~DQB1⁣*^*∗*^*06:02:01*, *protective*.

**Conclusions:** Idiopathic T1D is common among youth in Haiti. A significant proportion of all patients had preserved C-peptide secretion. Overall, predisposing and protective HLA patterns were identified. Study results highlight the importance of distinguishing T1D endotypes within and between populations.

## 1. Introduction

The incidence of youth-onset diabetes, which is predominantly type 1 diabetes (T1D), is increasing worldwide, particularly in low- and middle-income countries (LMICs) [1, 2]. In 1997, T1D was divided into immune-mediated T1D and idiopathic T1D [3]. The proportion of each form varies widely among the geographic populations. In white populations, which have been studied more extensively for T1D than other populations, the majority of people with T1D have the immune-mediated form, presenting with one or more pancreatic islet autoantibodies—glutamic acid decarboxylase antibody (GADA), insulin autoantibody (IAA), insulinoma-associated antigen-2 autoantibody (IA-2A), and zinc transporter 8 autoantibody (ZnT8A)—at disease onset [4, 5]. Non-white populations, including Asian, African, and other ethnic groups, are known to have a higher prevalence of autoantibody-negative diabetes in young people. Studies in the United Kingdom (involving a nonwhite group and testing GADA, IA-2A, and ZnT8A), Mali (testing GADA and IA-2A only), and India (testing GADA, IA-2A, and ZnT8A) showed autoantibody negativity in 27%, 31%, and 28% of young people with diabetes, respectively [6–8].

T1D is a highly heterogeneous disease in terms of clinical presentation, genetics, immunology, biology, histology, and therapeutic response. This complexity may be a limiting factor in T1D research and may explain the variable responses to therapeutic or preventive interventions. This has recently led to the conceptualization and categorization of T1D into endotypes (subtypes defined by distinct pathophysiological and functional mechanisms) rather than phenotypes [9, 10].

In Haiti, an LMIC of African descent, epidemiological data on youth-onset diabetes are scarce [11, 12], and there is virtually no published information on the impact of diabetes autoimmunity and genetics on this.

This study aimed to address these knowledge gaps by determining the metabolic characteristics of Haitian youth-onset diabetes and assessing whether islet cell autoantibody status is associated with specific patterns of clinical outcomes indicative of specific endotypes. We also aimed to identify predisposing and protective human leukocyte antigen (HLA) alleles and haplotypes in subjects diagnosed with T1D.

## 2. Methods

### 2.1. Study Site

This study was conducted by Fondation Haïtienne de Diabète et de Maladies Cardiovasculaires (FHADIMAC), Port-au-Prince, Haiti. Patients were recruited at the FHADIMAC, Kay Mackenson Clinic, and Diabetes Clinic at Milot Hospital in northern Haiti. Serological testing was performed at Bioendocrine Laboratory in Port-au-Prince, and HLA testing was performed at the Children's Hospital Oakland Research Institute (CHORI), USA. The study conformed to the Declaration of Helsinki; the protocol was approved by the National Bioethics Committee of the Haitian Ministry of Health and the CHORI Institutional Review Board. Verbal and written informed consent was obtained in Haitian Creole from subjects aged ≥18 years and from the parents or guardians of minors.

### 2.2. Study Subjects

This cross-sectional cohort study included participants with young onset insulin-treated diabetes. A total of 110 subjects aged ≤22 years, diagnosed with diabetes for <2 years, and treated with insulin were enrolled at the three centers between December 2014 and January 2018. Study participants were representative of regions across the country, including both rural and urban areas. All enrolled patients were considered to have T1D based on age at diagnosis, clinical features at presentation (e.g., diabetic ketoacidosis (DKA), polyuria, polydipsia, weight loss, and fatigue), high blood glucose (BG) levels at presentation, and a sustained need for insulin therapy within the first few weeks after diagnosis. HLA genotyping was successfully performed on 54 patients (30 males and 24 females randomly selected) and 66 healthy controls (23 males and 43 females) from the general population. Inclusion criteria for controls were aged ≥30 years, unrelated to another control subject, no diabetes, prediabetes, other autoimmune or endocrine diseases, random glycemia <85 mg/dL, no cell or organ transplant, no anticancer treatment, and no diabetes in first-degree relatives.

### 2.3. Demographic Data Collection

All patients completed a questionnaire collecting sociodemographic data, such as date of birth, sex, marital status, education level, occupation, and place of residence.

### 2.4. Clinical Parameters

Personal or family history was obtained from the medical records, patients, or families. First-degree family history of the disease, personal history of breastfeeding, height and weight trajectories, and thyroid and malabsorptive disorders were recorded. A history of malnutrition was defined as stunting, underweight, and/or wasting reported by the family or obtained from the medical records. Data on the family history of diabetes were also collected. Body weight, body fat, and fat-free mass were measured using a body composition analyzer in light clothing without shoes (SC-331S; TANITA Corporation of America, Inc., Arlington Heights, Illinois, USA). Height, weight, and waist and hip circumferences were measured using standard techniques. BMI *z*-scores were calculated using the 2000 Center for Disease Control (CDC) BMI-for-age growth charts for children aged 2–20 years [13]. For those aged >20 years, BMI *z*-scores were calculated using the age of 20 years. Subjects were classified as thin/underweight with *z*-scores <−2 SD, normal weight between −2 and + 0.99 SD, overweight between 1 and 1.99 SD, and obese ≥2 SD.

### 2.5. Sample Collection

Serum autoantibody and fasting C-peptide (FCP) measurements were performed within 5 days of blood collection. For HLA genotyping, ~200 μL of peripheral patient blood was preserved with DNAgard blood (Biomatrica, San Diego, CA, USA) and air-dried for storage and shipment. To supplement the DNA yield and improve quality, samples from 32 Haitian T1D individuals, including 25 originally collected individuals and seven previously uncollected T1D individuals, were collected and dried on GenSaver DNA blood spot cards (GenTegra, Pleasanton, CA). For the 200 control subjects, ~1 mL of saliva was mixed with 0.5 mL of DNAgard saliva-stabilizing reagent (Biomatrica, San Diego, CA, USA) for storage and shipment. Samples for T1D and control subjects were shipped to the research laboratory within 1 and 2 months, respectively.

### 2.6. Biochemical Parameters and Serology

Fasting plasma BG levels were measured in a fingerstick capillary blood sample using a Touch^TDM^ glucometer. FCP levels were measured using enzyme-linked immunosorbent assay (ELISA; IBL International, Hamburg, Germany). The normal range of the assay is 0.8–3.1 ng/mL. HbA_1_c levels were determined from capillary whole blood samples using a DCA Vantage device (Siemens Healthineers, Boston, MA, USA). DKA was defined as the presence of the clinical features of dyspnea, dehydration, altered consciousness associated with hyperglycemia (or marked glycosuria), and marked ketonuria (if urinalysis was performed). Information was mostly (>80%) obtained from medical records and, to a lesser extent, reported by the patients or their families. Serum samples were tested for three islet autoantibodies, GADA, IA-2A, and ZnT8A. GADA and IA-2A levels were determined using ELISA (IBL International, Hamburg, Germany) and were considered positive at levels >30 IU/mL. ZnT8A was determined using ELISA (Eagle Biosciences, Nashua, USA). The cutoff for a positive result was ≥15.0 IU/mL. Thyroid autoantibodies, thyroid peroxidase antibodies (TPO-Ab), and thyroglobulin antibodies (TG-Ab) were measured using a sequential sandwich ELISA (Monobind Inc., Lake Forest, USA). TPO-Ab and TG-Ab levels >40 and >125 IU/mL, respectively, were considered positive.

### 2.7. DNA Preparation and HLA Genotyping

*DNA preparation:* DNA was prepared from resuspended dried blood samples and stabilized saliva samples using QIAamp blood kits (QIAGEN, Germantown, MD) according to the manufacturer's instructions. The DNA elution buffer was preheated to 70°C, and the elution columns were incubated at 70°C for 10 min prior to centrifugation. Dried blood spots were removed from the cards using a 6 mm punch. DNA was recovered using GenSolve DNA Recovery kits (GenTegra, Pleasanton, CA, USA) and processed as described above.

*HLA genotyping:* Fifty-four T1D individuals and 66 controls were selected for HLA genotyping based on DNA quality and quantity, as assessed using a fragment analyzer (Agilent Technologies, Inc., Santa Clara, CA, USA). Sequencing libraries were prepared for all controls and five patients using the GenDx NGSgo-MX6-1 kit (*HLA-A*, *-B*, *-C*, *-DRB1*, *-DQB1*, and *-DPB1*) (GenDx, Utrecht, The Netherlands). Sequencing libraries for 51 T1D individuals were prepared using Holotype HLA 96/11 (*HLA-A*, *-B*, *-C*, *-DRB1*, *-DRB3*, *-DRB4*, *DRB5*, *-DQA1*, *-DQB1*, *-DPA1*, *-DPB1*) (Omixon Inc., Budapest, Hungary). One patient was included in both libraries. Sequencing was performed on the MiSeq platform (Illumina Inc., San Diego, CA, USA). Genotype calling for all samples was performed using two software packages: 1) Omixon HLA Twin version 4.2.0 (using IPD-IMGT/HLA Database release version 3.39.0 reference alignments) and 2) GenDx NGSengine version 2.23.1.23474 (using IPD-IMGT/HLA Database release version 3.45.1 alignments).

### 2.8. Statistical Analysis

Demographic and biochemical data were analyzed using Microsoft Excel (Redmond, USA). The mean differences between variables and correlations were calculated using an independent *t*-test and Pearson's correlation, respectively. Statistical analysis was conducted using IBM SPSS Statistics for Windows, Version 27.0. IBM Corporation, Armonk. Data were assessed for bimodality using likelihood ratio tests for nested finite mixture models. Models were fitted using an Expectation-Maximization algorithm implemented in R 3.3.1 (R Core Team, Vienna, Austria) and with the package “mix-tools” [14]. Significance was assessed using Wilks' chi-square approximation and parametric bootstrap with 1,000 samples. To assess the sensitivity to distributional assumptions, the analysis was performed in triplicate, assuming mixtures of normal, log-normal, and gamma populations. Graphs were created in Microsoft Excel.

BIGDAWG version 2.3.6 was used to perform locus-level (kx2) chi-squared (*χ*^2^) tests of heterogeneity and allele-level (2 × 2) *χ*^2^ tests of association between 54 T1D individuals and 66 control individuals at *HLA-A*, *HLA-B*, *HLA-C*, *HLA-DRB1*, *HLA-DQB1*, *and HLA-DPB1* loci at two-field (peptide) resolution [15]. BIGDAWG combines a set of alleles with expected counts <5 in either T1D individuals or control individuals that represent less than 20% of *χ*^2^ contingency table cells into a common “binned” category for analysis, as the *χ*^2^ test is invalid when >20% of contingency table cells have expected values <5 [15].

PyPop (v0.8.0) was used to test the Hardy–Weinberg equilibrium (HWE) proportions of HLA-DRB1 genotypes in T1D and control individuals [16]. Locus-level HWE deviations were tested using Guo and Thompson's [17] exact method. Chen's method was used to identify individual genotypes that significantly deviated from the HWE, with a significance threshold of 0.05 [18].

## 3. Results

Patient characteristics are shown in Table 1.

### 3.1. Demographic Data

**Figure 1:**
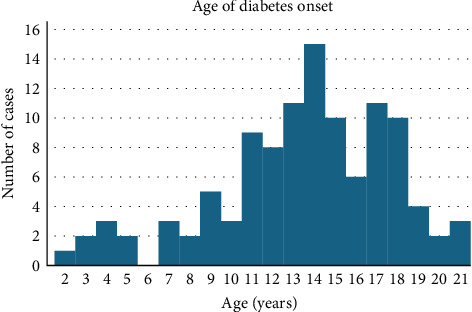
Age of diabetes onset.

Of the 110 patients, 60 (54.5%) were male and 50 (45.5%) were female (*p*=0.20). Age at diagnosis was 13.5 ± 4.2 years (range: 2–21 years) with a peak at 14 years. Age at evaluation was 14.5 ± 4.3 years (range: 3–22 years). Six participants (5.5%) were diagnosed at 0–4 years, 12 (10.9%) at 5–9 years, 46 (41.8%) at 10–14 years, 41 (37.3%) at 15–19 years, and 5 (4.6%) at 20–21 years (as shown in Figure 1).

### 3.2. Clinical and Biochemical Parameters

Sixteen (14.5%) first-degree relatives had a history of diabetes mellitus. A total of 100 patients (90.9%) had been breastfed for a median of 18 months (range: 2–28 months). Thyroid dysfunction was not reported. Clinical signs of malnutrition were reported in 10 (9.1%) patients. At diagnosis, the BG was 485 ± 160 mg/dL (27.0 ± 8.9 mmol/L) with a range of 120–700 mg/dL (6.7–38.9 mmol/L). One hundred and eight patients (98.2%) presented with at least one symptom of the classic triad of polyuria, polydipsia, and polyphagia, including 86 patients (78.2%) with all three symptoms. Weight loss was reported in 97 patients (88.2%), including two patients without triad symptoms. Eighteen (16.4%) patients presented with DKA.

At evaluation, the mean duration of diabetes was 11.7 ± 8.1 months. HbA1c was 10.6 ± 3.1% (92.0 ± 10.0 mmol/mol), with a range of 5.3–16.0% (34.0–151.0 mmol/mol). The mean FCP was 0.9 ± 1.0 ng/mL (normal range: 0.8–3.1 ng/mL). The FCP was low, normal, and high in 71 (64.6%), 35 (31.8%), and 4 (3.6%) patients, respectively. The mean BMI *z*-score was −0.9 ± 1.4. A BMI *z*-score of <−2 SD (underweight) was found in 21 of 108 patients (19.4%). Overall, the BMI *z*-score was higher in patients aged <14 years than in those aged ≥14 years (−0.56 ± 1.46 vs., −1.12 ± 1.29, *p*=0.03). This finding was related to the fact that in the autoantibody-negative group, patients aged <14 years had a higher BMI *z*-score than those aged ≥14 years (−0.29 ± 1.52 vs., −1.15 ± 1.18, *p*=0.01). No association was found between autoantibody positivity and BMI *z*-score.

FCP levels increased with BMI *z*-score regardless of islet autoimmunity status (0.6 ± 0.5 ng/mL, 0.8 ± 0.8 ng/mL, 1.9 ± 2.0 ng/mL, and 2.7 ± 2.0 ng/mL for underweight, normal weight, overweight, and obesity, respectively, *p*=0.0001). The mean daily insulin dose was 0.7 ± 0.3 U/kg. Thin patients had higher daily insulin dose requirements than patients with higher BMI z-scores, regardless of islet autoimmunity status (0.9 ± 0.2 U/kg vs., 0.7 ± 0.3 U/kg, *p*=0.03).

### 3.3. Autoantibody Results

Of the 110 patients tested, 48 (43.6%, 95% CI: 34.2–53.4) were positive for at least one islet autoantibody. Of the 48 autoantibody-positive patients, 30 were male (30/60, 50.0%) and 18 were female (18/50, 36.0%) (*p*=0.20).  Table 2 shows the autoantibody combinations in the 48 autoantibody-positive patients. Single positivity was observed in 41 patients (85.4%), including GADA in 27 (56.3%), ZnT8A in 8 (16.7%), and IA-2A in 6 (12.5%). Double and triple positivity were found in 3 (6.3%) and 4 (8.3%) autoantibody-positive patients, respectively. The addition of the ZnT8A assay to the GADA + IA-2A combination increased the rate of autoantibody positivity from 36.4% to 43.6%.

The autoantibody-negative patients were significantly older at diagnosis compared to the autoantibody-positive group (14.4 ± 3.5 years vs., 12.3 ± 4.8 years, *p*=0.01). ZnT8A was three times more frequent before than after 12 years of age (23.3% vs., 7.5%, *p*=0.04). None of the six patients diagnosed at 0–4 years of age were autoantibody-negative (*p*=0.06). About 3, 12, and 24 months after diagnosis, 38.9% (7/18), 51.4% (36/70), and 56.4% (62/110) of the patients evaluated were autoantibody-negative, respectively. The autoantibody-negative patients were more likely not to have breastfed than autoantibody-positive patients (*p*=0.04). Infections were more common in autoantibody-negative patients than in autoantibody-positive patients at diagnosis, regardless of BG level at presentation (20/62 vs., 5/48, *p*=0.01). The same was true for visual impairment (29/62 vs., 11/48, *p*=0.02). Forty-two patients had low FCP levels (42/71, 59.2%), 16 patients had normal FCP levels (16/35, 45.7%), and all patients with high FCP levels (4/4, 100%) were autoantibody-negative.

TPO-Ab and TG-Ab were positive in 1.1% and 2.2% of the patients, respectively.

After adjustment for the covariates age at diagnosis, BMI z-score, sex, duration of diabetes, history of breastfeeding, and malnutrition, only younger age at diagnosis (OR: 0.87; CI 95%: 0.78–0.97; *p*=0.013) and history of breastfeeding (OR: 12.33; CI 95%: 1.29–118.15; *p*=0.029) were significantly associated with the presence of one or more islet autoantibodies.

### 3.4. HLA Allele and Haplotype Analyses

HLA genotyping data were generated for 54 T1D patients and 66 healthy controls. Whole gene sequencing data were generated for all classical HLA loci (*HLA-A*, *-B*, *-C*, *-DRB1*, *-DRB3*, *-DRB4*, *-DRB5*, *-DQA1*, *-DQB1*, *-DPA1*, and *-DPB1*) in T1D individuals and for a subset of six loci (*HLA-A*, *-B*, *-C*, *-DRB1*, *-DQB1*, and *-DPB1*) in control individuals because of resource limitations. Disease association analyses were performed for a subset of six loci as well as the haplotype subsets of these loci. Data were generated at the highest resolution (four-field) level; however, association analyses were limited to coding sequences (three-field), excluding introns and untranslated sequences.

No significant locus-level association was observed for any of the HLA class I loci (*HLA-A*, *-B*, *-C*). Marginally significant allele-level associations for *HLA-B*⁣*^*∗*^18:01:01* (OR = 5.06, *p*=0.03) and *-B*⁣*^*∗*^58:02:01* (OR = 0.22, *p*=0.04) alleles did not withstand correction for multiple comparisons. No further class I allele-based analysis was performed.

For HLA class II loci, both *HLA-DRB1* (*p*=0.005) and -*DQB1* (*p*=0.0002), but not *DPB1*, showed locus-level significance after correction for multiple comparisons. The HLA class II haplotypes *DRB1~DQB1*, *DRB1~DQB1~DPB1*, and *DRB1~DPB1* were significantly associated after correction.

Table 3 shows HLA class II alleles and haplotypes significantly associated with T1D. For *DRB1* alleles, these included *DRB1⁣*^*∗*^*03:01:01* (OR = 4.27, *p*=0.002) and *DRB1*⁣*^*∗*^09:01:02* (OR = 3.60, *p*=0.012), both predisposing; *DRB1*⁣*^*∗*^08:04:01* (OR = 0.27, *p*=0.036) and *DRB1*⁣*^*∗*^15:03:01* (OR = 0.44, *p*=0.029), both protective. T1D-associated *DQB1* alleles included DQB1*⁣*^*∗*^02:01:01 (OR = 3.63, *p*=0.003) and DQB1*⁣*^*∗*^02:02:01 (OR = 2.90, pc = 0.003), both predisposing; and DQB1*⁣*^*∗*^06:02:01 (OR = 0.34, *p*=0.002), protective. Notably, the binned category for *DQB1* is protective in these data (OR = 0.39, *p*_c_ = 0.019). Three *DRB1~DQB1* haplotypes were strongly associated with T1D: *DRB1*⁣*^*∗*^03:01:01~DQB1*⁣*^*∗*^02:01:01* (OR = 4.27, *p*=0.002), *DRB1*⁣*^*∗*^09:01:02~DQB1*⁣*^*∗*^02:02:01* (OR = 4.54, *p*=0.005), both predisposing; and *DRB1*⁣*^*∗*^15:03:01~DQB1*⁣*^*∗*^06:02:01* (OR = 0.35, *p*=0.009), protective. A complete list of the 51 unique DRB1~DQB1 haplotypes observed in the data can be found in Table S1. Only 6 of 51 were present in sufficient frequency to be analyzed independently; these six did not include any with the protective allele *DRB1*⁣*^*∗*^08:04:01*, which was observed in haplotype with five different DQB alleles in the data set.

Although no allele at the *DPB1* locus was significantly associated with T1D on its own, the addition of this locus to the protective *DRB1*⁣*^*∗*^15:03:01~DQB1*⁣*^*∗*^06:02:01* haplotype can have different susceptibility effects. Indeed, the combination *DRB1*⁣*^*∗*^15:03:01~DQB1*⁣*^*∗*^06:02:01~DPB1*⁣*^*∗*^02:01:02*, observed in 13 controls and no cases, enhanced the protective effect, showing very strong protection (OR = 0.00,pc = 0.001), while *DRB1*⁣*^*∗*^15:03:01~DQB1*⁣*^*∗*^06:02:01~DPB1*⁣*^*∗*^01:01:01*, observed in five cases and five controls, showed no significant association.

**Figure 2:**
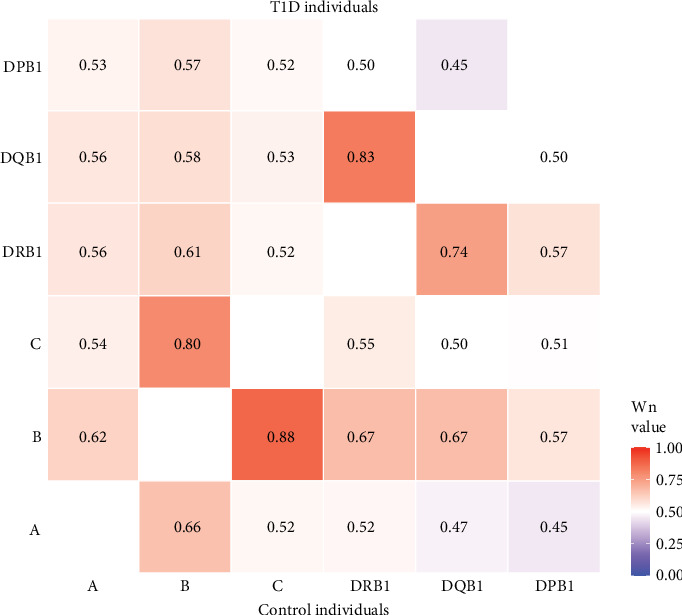
Pairwise matrix of *W*_n_ values in Haitian T1D individuals and control Individuals. Pairwise *W*_n_ values for T1D individuals are shown in the upper half of the matrix, while *W*_n_ values for control individuals are shown in the lower half. As show in the inset scale, the range of values is color coded from red to blue. T1D, type 1 diabetes.

As illustrated in Figure 2, extensive linkage disequilibrium (LD) was observed between the *HLA-DRB1* and -*DQB1* loci and between the *HLA-B* and *-C* loci, with higher *W*_*n*_ values observed between the *HLA-DRB1* and -*DQB1* loci in T1D individuals and higher *W*_*n*_ values between the *HLA-B* and *-C* loci in control individuals. This pattern is consistent with the observed T1D-association for *DRB1~DQB1* haplotypes, but not with class I loci in Haitian T1D individuals.

## 4. Discussion

This study is the first to investigate T1D endotypes in youth in Haiti in detail, including islet autoantibodies and HLA status.

### 4.1. Considerations on the High Prevalence of Idiopathic Diabetes

We found that more than half of our patient cohort was autoantibody-negative, which is a much higher rate than that observed in populations of European descent. The amplitude of this prevalence has several possible explanations. First, there was the 1-year delay between diagnosis and antibody testing. Second, there is the nontesting of IAA with the traditional immunoassays, which cannot be used after 2 weeks of insulin treatment. From the study data, we cannot estimate the impact of the lack of AAI screening on the overall prevalence of islet autoantibodies. However, many publications on the subject suggest a low diagnostic sensitivity and cost-effectiveness of IAA in older children and adults after diabetes onset. For example, Williams et al. [19] found that IAA positivity was negatively associated with an age at onset >11 years. Redondo et al. [20], analyzing the data from the Type 1 Diabetes TrialNet Pathway to Prevention study, showed that the IAA positivity at diagnosis in older children and adults is most often associated with one or more other islet autoantibodies. Other explanatory factors include the possible presence of autoantibodies to unidentified or minor autoantigens, the weakness or temporary absence of a humoral response in some patients with autoimmune T1D, and the possible existence of a high prevalence of a subtype of diabetes with an aetiopathogenic mechanism different from that of T1-immune-mediated diabetes, with or without distinct phenotypes. The latter possibility is suggested by recent observations of two distinct endotypes related to the age of diabetes onset in high-income countries [21]. Indeed, using data from the Finnish Pediatric Diabetes Register, Parviainen et al. [21] found that children diagnosed with diabetes at <7 years of age had a hyperimmune insulitis pattern characterized by more aggressive clinical phenotypes, whereas those diagnosed at ≥13 years of age had a pauci-immune insulitis pattern characterized by more beta cells with residual endogenous insulin secretion, higher blood C-peptide levels, and lower proinsulin to C-peptide ratios. Notably, in our study, autoantibody-negative diabetes was diagnosed at a significantly older age than autoantibody-positive diabetes (14.4 years vs., 13.5 years), and none of the patients in the youngest age group of 0–4 years were autoantibody-negative. In addition, more than one-third of C-peptide levels were not low, which was not related to patient age. Palmer [22], comparing several studies on the evolution of C-peptide levels in T1D, showed that its decline during the first year was highly variable. Therefore, our findings place our study sample, whose average duration of diabetes was 11.7 months, in the group with a high proportion of patients with preserved C-peptide secretion.

Among the autoantibody-positive subjects, GADA was the most common T1D-associated autoantibody, followed by ZnT8A and IA2-A. This pattern is similar to that found in established T1D cases (duration >12 months) in a scoping review by Ross et al. [23]. The addition of ZnT8A to the GADA/IA-2A combination increased the detection rate of autoimmune T1D by 7.3%. ZnT8A was marginally more common in younger patients. However, no conclusions can be drawn from this finding, which is borderline statistically significant. In addition, studies on this topic do not seem to be consistent. For example, a study in Poland showed a higher prevalence and titer of ZnT8A in children than in adults with newly diagnosed T1D [24], while in a Finnish study of 2115 subjects aged <15 years, ZnT8A positivity was associated with older age at T1D diagnosis [25].

Thus, our study population comprised of at least two T1D endotypes. Approximately half of the patients were autoantibody-negative, with older age at diabetes onset and higher BMI *z*-scores in younger patients. The other half were antibody-positive, with a younger age of onset, and no correlation with the BMI *z*-score. Nevertheless, the mean total insulin dose per kilogram was similar between the two groups. Prospective, long-term observational studies with larger numbers of participants, pancreatic immunohistological sampling, and genetic correlations are needed to further evaluate our hypothesis regarding endotypes. The recent review by Weston et al. [10] can be used to guide the design of such studies.

### 4.2. Sex Distribution of T1D Autoimmunity

Our data showed no sex predominance in the prevalence of either T1D type. Worldwide, there appears to be a male predominance in youth-onset T1D in countries with a higher incidence [26] and a female predominance in countries with a lower incidence [27]. Larger registry-based national data are needed to confirm our findings and to monitor sex distribution and trends in incidence and prevalence over time.

### 4.3. Thyroid Autoimmunity and T1D

T1D patients are known to be at increased risk of thyroid autoimmunity. Indeed, thyroid autoantibodies are common in children and adolescents with T1D, and autoimmune thyroid disease (AITD), mainly Hashimoto thyroiditis and Basedow disease, is the most common T1D-associated autoimmune disorder. In a review by Kakleas et al. [28], the prevalence of thyroid autoantibody positivity in patients with T1D was 12.1%−23.4%; clinical Hashimoto's hypothyroidism and Grave's disease were found in 4%–18% and 1.5%–4% of the patients, respectively. More recently, in another review, Popoviciu et al. [29] reported a 17%–30% prevalence of AITD in patients with T1D. This variation in the prevalence of the thyroid disease may be due to the fact that the rate of thyroid autoantibodies increases with age and duration of diabetes. Indeed, some studies have shown a peak in the prevalence of thyroid autoimmunity around puberty and 3.5–4 years after diabetes diagnosis [26]. In our study, we found a very low prevalence of thyroid autoantibodies, suggesting a lack of association between Haitian patients with T1D and thyroid autoimmunity. However, the small sample size and the short duration of diabetes may also explain this. Further studies in additional Haitian cohorts and prospective longitudinal monitoring, including a national T1D registry, are recommended, given the immediate clinical implications, such as the need to monitor thyroid function in Haitian patients with T1D.

### 4.4. Beta Cell Function and Metabolic Considerations

Mean HbA1c was very high in our study population, which is consistent with recent reports from other resource-limited settings. In a study conducted in Kenya, Ngwiri et al. [30] found a median HbA1c of 12.1% in patients with T1D aged 1–19 years.

Mean HbA1c of 10.4%, 9.6%, and 11.1%, was reported in studies of children and adolescents with T1D in Sudan, Ethiopia, and Tanzania, respectively [31–33]. Social determinants, including socioeconomic status, education, and access to quality health care and insulin, are almost ubiquitously unfavorable in Haiti. They are most likely the main drivers of chronic hyperglycemia. The aforementioned slower decline in beta cell function with residual, albeit low, endogenous insulin secretion in our patients compared to European patients may be a protective factor against ketosis but not hyperglycemia. Indeed, in a setting where optimal diabetes management and access to insulin are challenging, residual beta cell function may increase the risk of exposure to prolonged chronic hyperglycemia without acute decompensation (DKA). This may explain the relatively low DKA rate of 16.3% in our study compared with the rates of 12.8% to 80% reported by Usher-Smith et al. [34] in a systematic review of country variations in the frequency of DKA in children. However, two limiting factors need to be considered. First, the methods used to estimate the prevalence of DKA at the time of diagnosis may often result in underestimation. Second, low awareness of hyperglycemia and DKA symptoms in this resource-limited setting may lead to a missed diagnosis of these conditions, resulting in premature death from DKA before diagnosis, especially in young children. An 8-year community-based DKA awareness intervention in Italy involving children aged 6–14 years with new-onset T1DM, teachers, pediatricians, and parents reduced the incidence of DKA from 78% to 12.5% [35, 36]. Therefore, an awareness campaign on the clinical signs of T1D and DKA in Haiti may enable timely diagnosis of these two related conditions, thereby reducing associated morbidity and mortality. Another consideration is that the combination of above-target HbA1c and a low rate of DKA represents a specific phenotype of diabetes in young people in Haiti, regardless of autoimmune status, whereby residual endogenous insulin levels are sufficient to suppress ketosis but insufficient to maintain euglycemia.

### 4.5. Commonalities and Differences of T1D-Related Alleles With Other Populations

Despite the large number of studies on the contribution of HLA to T1D risk, there are very few reports on Caribbean populations, with the extent of admixture varying among populations [37–39]. The population of Haiti is reported to be 90%–95% African in origin, with little contribution from other ethnicities [40, 41]. The HLA data presented here are consistent with this, revealing the presence and T1D association of alleles such as *DRB1*⁣*^*∗*^15:03:01*, commonly found in African populations, but not in European or Asian populations.

HLA associations observed in this dataset are generally consistent with those reported for HLA-associated T1D risk in other populations [42, 43]. For example, *DRB1*⁣*^*∗*^03:01:01* is a risk allele for T1D in nearly all populations studied, and *DRB1*⁣*^*∗*^15:xx* alleles (*⁣*^*∗*^15:01, *⁣*^*∗*^15:02, and *⁣*^*∗*^15:03, observed in European, Asian, and African populations, respectively) are protective. Although multiple alleles in the group commonly referred to as “DR4” (including *DRB1*⁣*^*∗*^04:01/04:02/04:04/04:05* and excluding *DRB1*⁣*^*∗*^04:03/*⁣*^*∗*^04:06*) demonstrate positive association with T1D in many populations, particularly those of European descent, no significant T1D association was observed for any DR4 allele or haplotype in these data. This observation may reflect a stronger association for DR3 than for DR4 in this population, or it may simply reflect a lower frequency for DR4 than for DR3 in this population and the limited size of the study. More data are needed to address this issue.

HLA class II is more strongly associated with T1D than HLA class I; however, class I associations are frequently observed. The lack of significant class I associations in these data is supported by LD results, which showed a stronger LD between DRB1 and DQB1 in T1D individuals than in controls and a stronger LD between HLA-B and HLA-C in controls than in T1D individuals.

The lack of class I associations in this dataset may reflect a difference in the Haitian population from other populations, or may be attributable to the small sample size. The observation that the protective haplotype *DRB1*⁣*^*∗*^15:03:01~DQB1*⁣*^*∗*^06:02:01* can be stratified by *DPB1* into two haplotypes, *DRB1*⁣*^*∗*^15:03:01~DQB1*⁣*^*∗*^06:02:01~DPB1*⁣*^*∗*^01:01:01 and DRB1*⁣*^*∗*^15:03:01~DQB1*⁣*^*∗*^06:02:01~DPB1*⁣*^*∗*^02:01:02*, with very different T1D susceptibility effects, is novel and unexpected. However, *DPB1*⁣*^*∗*^02:01:02* is not considered to be a T1D risk allele. This suggests that the added protection from *DPB1*⁣*^*∗*^02:01:02* may have resulted from haplotype-derived factors other than the allele itself. Finally, the protective effect of the binned category for *DQB1* in these data may be attributable to the fact that a high proportion of binned DQB1 alleles exhibited protective effects in other populations (e.g., DQB1*⁣*^*∗*^03:01:04) or were in haplotype combination with DRB1 alleles that are generally protective (e.g., DRB1*⁣*^*∗*^08:04 and DRB1*⁣*^*∗*^11:01).

### 4.6. Study Limitations

Our study has several limitations. The sample sizes were small and may have prevented us from finding certain significant associations, particularly between T1D and HLA class I, and from comparing the relationship between HLA and immune versus nonimmune T1D due to reduced statistical power. The performance of pancreatic autoantibody assays varies between ethnic populations. Our study did not use population-specific thresholds for T1D-associated autoantibody levels. Several variables were reported by the patient or family, which may have introduced a bias. FCP has limitations in terms of performance (sensitivity and reproducibility) compared with stimulated C-peptide tests, especially the mixed meal tolerance test (MMTT) and the post-glucagon stimulation test (GST) [44, 45]. However, it remains the best we can do. On the other hand, the study has two important strengths. For the first time, we have clinical, immunological, and genetic data on more than 100 youths with diabetes in Haiti. In addition, we obtained significant results that underline the importance of distinguishing endotypes, possibly by population type.

## 5. Conclusion

This study is the first to provide a detailed description of the clinical presentation, autoimmune status, and HLA genetics of Haitian youth with diabetes. The results highlight the clinical and epidemiological importance of distinguishing T1D endotypes within and between populations. We highlight previously unknown predisposing and protective HLA patterns and draw attention to the large number of nonautoimmune-positive T1D cases, especially in older Haitian youth, and the large number of FCP-positive patients. In a resource-limited setting, where chronic hyperglycemia is not recognized quickly or is not adequately addressed, the benefits that patients typically derive from prolonged beta cell function may be mitigated. Prospective longitudinal research in additional cohorts, ideally using a national registry, is needed to further confirm our findings and draw more definitive conclusions for clinical care. Comparative research with European and other non-European descent populations would help to further elucidate endotype differences and their implications for clinical practice and patient outcomes.

## Figures and Tables

**Table 1:** Characteristics of patients (*n* = 110).

	All patients	Islet antibody-positive and negative patients				
		iAb+ (*n* = 48)	iAb− (*n* = 62)	OR	95% CI	*p* value
Gender						
Female	50 (45.5)	18 (36.0)	32 (64.0)	0.6	0.3–1.2	0.20
Male	60 (54.5)	30 (50.0)	30 (50.0)	—	—	—
Age at diagnosis, years	13.5 ± 4.2	12.3 ± 4.8	14.4 ± 3.5	—	—	0.01
Family history (first-degree relatives)						
Diabetes	16 (14.5)	6 (12.5)	10 (16.1)	0.7	0.3–2.2	0.79
Personal history						
Breastfeeding	100 (90.9)	47 (97.9)	53 (85.5)	8.0	1.0–357.1	0.04
Breastfeeding duration (months)	18 [11–19]	17 [7–19]	18 [13–22]	—	—	0.05
Thyroid disorder	0 (0.0)	0 (0.0)	0 (0.0)	—	—	—
Goiter	0 (0.0)	0 (0.0)	0 (0.0)	—	—	—
Malnutrition	10 (9.1)	1 (2.1)	9 (14.5)	0.1	0.0–1.0	0.04
At diagnosis						
Glycemia	485 ± 160	455 ± 161	510 ± 156	—	—	0.10
Polyuria	105 (95.5)	46 (95.8)	59 (95.2)	1.2	0.2–7.3	1.00
Polydipsia	96 (87.3)	39 (95.8)	57 (91.9)	0.4	0.1–1.2	0.17
Polyphagia	89 (80.9)	36 (75.0)	53 (85.5)	0.5	0.2–1.3	0.25
Weight loss	97 (88.2)	44 (91.7)	53 (85.5)	1.9	0.5–6.5	0.38
Fatigue	70 (63.6)	26 (54.2)	44 (71.0)	0.5	0.2–1.1	0.11
Sleepiness	63 (57.3)	23 (47.9)	40 (64.5)	0.5	0.2–1.1	0.12
Infection	25 (22.7)	5 (10.4)	20 (32.3)	0.2	0.1–0.7	0.01
Skin pruritus	18 (16.4)	9 (18.8)	9 (14.5)	1.4	0.5–3.7	0.74
Vaginal pruritus (females)	23 (46.0)	9 (50.0)	14 (43.8)	1.3	0.4–4.1	0.9
Visual disturbance	40 (36.4)	11 (22.9)	29 (46.8)	0.3	0.2–0.8	0.02
DKA	18 (16.4)	5 (10.4)	13 (21.0)	0.4	0.1–1.3	0.22
At evaluation						
Age at evaluation, years	14.5 ± 4.3	13.2 ± 4.7	15.4 ± 3.8	—	—	0.01
Diabetes duration, months	11.7 ± 8.1	10.2 ± 7.7	12.9 ± 8.3	—	—	0.08
Hyperglycemic crisis	41 (37.3)	14 (29.2)	27 (43.5)	0.5	0.2–1.2	0.18
Severe hypoglycemia	14 (12.7)	7 (14.6)	7 (11.3)	1.3	0.4–4.1	0.82
Frequent hypoglycemia	12 (10.9)	7 (14.6)	5 (8.1)	2.0	0.6–6.6	0.44
All-cause hospitalization	84 (76.4)	33 (68.8)	51 (82.3)	0.5	0.2–1.2	0.15
HbA1c, %	10.6 ± 3.1	11.1 ± 3.3	10.1 ± 2.8	—	—	0.11
HbA1c, mmol/mol	92.0 ± 10.0	98.0 ± 12.0	87.0 ± 7.0	—	—	0.11
Fasting C-peptide	0.9 ± 1.0	0.8 ± 0.6	0.9 ± 1.3	—	—	0.53
<0.8 ng/mL (<0.26 nmol/L)	71 (64.6)	29 (60.4)	42 (67.7)	0.7	0.3–1.6	0.55
0.8–3.1 ng/mL (0.26–1.03 nmol/L)	35 (31.8)	19 (39.6)	16 (25.8)	1.9	0.8–4.2	0.18
≥3.1 ng/mL (>1.03 nmol/L)	4 (3.6)	0 (0.0)	4 (6.5)	0.0	—	0.20
BMI z-score	−0.9 ± 1.4	−0.9 ± 1.5	−0.8 ± 1.4	—	—	0.72
Underweight (<−2)	21 (19.4)	12 (57.1)	9 (42.9)	1.9	0.7–4.9	0.28
Normal (−2.00−0.99)	77 (71.3)	32 (41.6)	45 (58.4)	0.7	0.3–1.7	0.56
Overweight (1.00–1.99)	7 (6.5)	3 (48.9)	4 (57.1)	0.9	0.2–4.4	1.00
Obese (≥2.00)	3 (2.8)	1 (33.3)	2 (66.7)	0.6	0.1–7.0	1.00
Body fat, kg	6.5 ± 4.8	5.8 ± 4.0	7.0 ± 5.2	—	—	0.20
Body free fat mass, kg	37.1 ± 11.7	35.4 ± 13.0	38.5 ± 10.4	—	—	0.18
Waist-to-hip ratio						
Female	0.9 ± 0.1	0.9 ± 0.1	0.9 ± 0.1	—	—	0.76
Male	0.9 ± 0.1	0.9 ± 0.1	0.9 ± 0.1	—	—	0.65
Daily insulin dose, U/kg	0.7 ± 0.3	0.8 ± 0.3	0.7 ± 0.3	—	—	0.83
TG-Ab positive	2 (1.9)	0 (0.0)	2 (3.3)	0.0	—	0.50
TPO-Ab positive	1 (0.9)	0 (0.0)	1 (1.7)	0.0	—	1.00

*Note:* Values are presented as the number of patients (percentages), means (± SD), or median [interquartile ranges].

**Table 2:** Islet autoantibodies (IAb) and their combinations among patients.

Islet autoantibodies	*N* (%)	95% CI
≥ 1 IAb+ (*n* = 110)	48 (43.6%)	34.2–53.4

**Islet autoantibodies combinations (*n* = 48)**	***N***	**%**

Single IAb+	41	85.40%
GADA+	27	56.30%
IA-2A+	6	12.50%
ZnT8A+	8	16.70%
2 IAb+	3	6.30%
GADA + and IA-2A+	2	4.20%
GADA + and ZnT8A+	1	2.10%
IA-2A+ and ZnT8A+	0	0.00%
3 IAb+	4	8.30%
Any IAb+		
Any GADA+	34	70.80%
Any IA-2A+	12	25.00%
Any ZnT8A+	13	27.10%
GADA + and/or IA-2A+	40	83.30%
GADA + and/or ZnT8A+	42	87.50%
IA-2A+ and/or ZnT8A+	21	43.80%

**Table 3:** Significant associations observed in the dataset.

Locus or haplotype^a^	Allele (s)	Cases *n* (freq)	Controls *n* (freq)	OR (95%CI)	*p* value
DRB1	All	—	—	10.00 (0.00-*⁣*^*∗*^)	0.0083
DQB1	All	—	—	11.00 (0.00-*⁣*^*∗*^)	0.0083
DRB1	3:01:01	19 (0.18)	6 (0.05)	4.27 (1.55–13.53)	0.0015
DRB1	8:04:01	3 (0.03)	12 (0.10)	0.27 (0.05–1.05)	0.0357
DRB1	9:01:02	14 (0.13)	5 (0.04)	3.60 (1.17–13.19)	0.0120
DRB1	15:03:01	11 (0.10)	26 (0.21)	0.44 (0.18–0.98)	0.0290
DQB1	2:01:01	19 (0.18)	7 (0.06)	3.63 (1.38–10.62)	0.0035
DQB1	2:02:01	27 (0.25)	13 (0.10)	2.90 (1.34–6.48)	0.0029
DQB1	6:02:01	13 (0.12)	36 (0.29)	0.34 (0.16–0.71)	0.0019
DQB1	Binned	9 (0.08)	24 (0.19)	0.39 (0.15–0.92)	0.0189
DRB1~DQB1	03:01:01–02:01:01	19 (0.18)	6 (0.05)	4.27 (1.55–13.53)	0.0015
DRB1~DQB1	09:01:02–02:02:01	14 (0.13)	4 (0.03)	4.54 (1.36–19.45)	0.0051
DRB1~DQB1	15:03:01–06:02:01	9 (0.08)	26 (0.21)	0.35 (0.14–0.82)	0.0085
DRB1~DQB1	Binned	46 (0.43)	72 (0.57)	0.56 (0.32–0.97)	0.0265
DRB1~DQB1~DPB1	09:01:02–02:02:01–01 : 01:01	9 (0.08)	3 (0.02)	3.73 (0.89–21.85)	0.0396
DRB1~DQB1~DPB1	15:03:01–06:02:01–02:01:02	0 (0.0)	13 (0.10)	0.00 (0.00–0.36)	0.0006
DRB1~DPB1	15:03:01–02:01:02	0 (0.0)	10 (0.08)	0.00 (0.00–0.50)	0.0028

*Note:* Frequencies are given with numbers of T1D individuals (cases) and numbers of individuals without diabetes (controls). Odds ratios (OR) are given with the boundaries of the 95% confidence intervals (CI).

^a^Frequency and count values for all DRB1~DQB1 haplotypes are provided in Table S1.

## Data Availability

The data that support the findings of this study are available from the corresponding author, [E.J.B.], upon reasonable request.

## Supporting Information

Supporting Information The supplement contains a single table listing the 51 DRB1~DQB1 haplotypes observed in the data set and indicates the six haplotypes that were present in sufficient frequency for individual analysis.
