# Supplementary material for: Etiologic Determinants and Characteristics of Diabetes in Haitian Youth (EDDHY Study)
Source: Pediatr Diabetes. 2025 May 25;2025:9974561. doi: 10.1155/pedi/9974561 (PMC12127126; doi:10.1155/pedi/9974561)
Supplement: Supporting Information — The supplement contains a single table listing the 51 DRB1~DQB1 haplotypes observed in the data set and indicates the six haplotypes that were present in sufficient frequency for individual analysis. [file 9974561.f1.docx]

**Supplementary Table 1.**  All DRB1~DQB1 haplotypes observed in the data set. Six haplotypes with sufficient frequency for individual analysis are marked with *.

| **DRB1~DQB1** | **cases n (freq)** | **controls n (freq)** |
| --- | --- | --- |
| 01:01:01~05:01:01 | 4 (0.037) | 0 (0) |
| 01:02:01~05:01:01 | 1 (0.009) | 6 (0.048) |
| 01:02:13~05:01:01 | 7 (0.065) | 0 (0) |
| *03:01:01~02:01:01 | 19 (0.176) | 6 (0.048) |
| 03:02:01~02:02:01 | 1 (0.009) | 0 (0) |
| 03:02:01~04:02:01 | 3 (0.028) | 5 (0.04) |
| 03:102~04:02:01 | 0 (0) | 1 (0.008) |
| 04:01:01~03:19:01 | 1 (0.009) | 0 (0) |
| 04:02:01~03:02:01 | 0 (0) | 1 (0.008) |
| 04:05:01~02:02:01 | 0 (0) | 1 (0.008) |
| *04:05:01~03:02:01 | 6 (0.056) | 4 (0.032) |
| 04:08:01~05:02:01 | 0 (0) | 1 (0.008) |
| *07:01:01~02:02:01 | 10 (0.093) | 8 (0.063) |
| 07:01:01~03:03:02 | 0 (0) | 1 (0.008) |
| 08:01:01~04:02:01 | 1 (0.009) | 0 (0) |
| 08:04:01~02:01:01 | 0 (0) | 1 (0.008) |
| 08:04:01~03:01:04 | 2 (0.019) | 6 (0.048) |
| 08:04:01~03:19:01 | 1 (0.009) | 2 (0.016) |
| 08:04:01~04:02:01 | 0 (0) | 2 (0.016) |
| 08:04:01~04:41N | 0 (0) | 1 (0.008) |
| 08:06~03:01:01 | 0 (0) | 2 (0.016) |
| 08:06~06:02:01 | 0 (0) | 2 (0.016) |
| *09:01:02~02:02:01 | 14 (0.13) | 4 (0.032) |
| 09:01:02~06:02:01 | 0 (0) | 1 (0.008) |
| 10:01:01~05:01:01 | 1 (0.009) | 3 (0.024) |
| 11:01:02~03:01:01 | 1 (0.009) | 2 (0.016) |
| 11:01:02~03:19:01 | 1 (0.009) | 0 (0) |
| 11:01:02~05:02:01 | 0 (0) | 3 (0.024) |
| *11:01:02~06:02:01 | 4 (0.037) | 6 (0.048) |
| 11:02:01~03:01:01 | 1 (0.009) | 2 (0.016) |
| 11:02:01~03:19:01 | 1 (0.009) | 3 (0.024) |
| 11:02:01~03:243 | 1 (0.009) | 0 (0) |
| 11:04:01~03:01:01 | 0 (0) | 2 (0.016) |
| 12:01:01~03:01:01 | 1 (0.009) | 1 (0.008) |
| 12:01:01~05:01:01 | 1 (0.009) | 3 (0.024) |
| 13:01:01~03:03:02 | 1 (0.009) | 1 (0.008) |
| 13:01:01~05:01:01 | 5 (0.046) | 2 (0.016) |
| 13:01:01~06:03:01 | 0 (0) | 5 (0.04) |
| 13:02:01~03:01:01 | 0 (0) | 1 (0.008) |
| 13:02:01~05:01:01 | 2 (0.019) | 3 (0.024) |
| 13:02:01~06:04:01 | 0 (0) | 2 (0.016) |
| 13:02:01~06:09:01 | 1 (0.009) | 1 (0.008) |
| 13:03:01~02:02:01 | 1 (0.009) | 0 (0) |
| 13:03:01~03:01:01 | 2 (0.019) | 0 (0) |
| 13:04~05:01:01 | 0 (0) | 1 (0.008) |
| 13:16:01~06:04:01 | 1 (0.009) | 1 (0.008) |
| 15:01:01~06:02:01 | 0 (0) | 1 (0.008) |
| 15:03:01~02:02:01 | 1 (0.009) | 0 (0) |
| *15:03:01~06:02:01 | 9 (0.083) | 26 (0.206) |
| 15:03:01~06:09:01 | 1 (0.009) | 0 (0) |
| 16:02:01~05:02:01 | 2 (0.019) | 2 (0.016) |
